# Buboes: A Warning

**Published:** 1909-06-05

**Authors:** 


### Surgery.

### BUBOES: A WARNING.

As buboes are commonly supposed to represent a condition, or, perhaps, better, a complication, devoid of any special danger to life, and as the surgical operative treatment of such of them as may require it is classed under the head of minor surgery, it is perhaps natural that little or no emphasis has been laid on the dangers which may, and not infrequently do, result in connection with this branch of surgery. Professor Eugene Fuller, of New York, has published some cases illustrative of the catastrophes that have arisen in different patients after operations upon buboes, and he has emphasised the necessity of treating buboes with circumspection.

Buboes generally lie just above Poupart's ligament, less commonly below it, and sometimes over it. In cases of active suppuration a fairly extensive area both above and below it is frequently involved. The deep limits of such suppurations are determined by the external oblique layer of the abdominal fascia, Poupart's ligament, and the deep fascia covering Scarpa's triangle. Over these structures Nature, if left to herself, throws out a barrier of cellular infiltration and granulations, which, as a rule is effective in protecting the important parts beneath from invasion by the suppurative process. Such suppurations, thus checked from extending in a deep direction, tend to burrow subcutaneously and to point externally. Just below the abdominal fascia is the important lymph space, limited externally by the abdominal fascia and the fascia propria of the pelvis, and internally by the peritoneum. In this lymph space lie the external iliac artery and vein, while between the layers of the fasciæ, and still more exposed, are the superficial epigastric and circumflex iliac vessels. The femoral artery and vein lie in Scarpa's triangle, just under the fascia, and, consequently, much nearer to the surface than are the external iliac vessels.

The dangers in connection with the surgery of buboes lie in two directions. First, in a direct or indirect injury to the blood-vessels just mentioned, and, second, in an inflammatory invasion of the lymph space between the fascia propria of the pelvis and the peritoneum, the infection having entered through the abdominal fascia. Direct injury to blood-vessels lies in an actual wound, during operation, of the femoral artery or vein, of the external iliac artery or vein, or of the epigastric or circumflex iliac artery or vein. Cutting into branches of the main vessels is less serious at the time, but there is still the risk of the purulent infection loosening the ligature, with secondary hæmorrhage as the result.

The danger from indirect injury to these blood vessels lies in unduly exposing their walls during operation through removal of the connective tissue and the fascial coverings, thus allowing the infective process causing the bubo to come into direct contact with them. Infection coming into such direct contact with these blood-vessels may cause septic phlebitis, or, through ulcerative action, secondary arterial hæmorrhage.

Inflammatory invasion of the deep lymph space mentioned above is generally occasioned by two surgical errors. First, at the time of operation the basement structures of the bubo are so removed by curettage, or by cutting with curved scissors, that the abdominal fascia is laid bare and perhaps actually damaged. The second error lies in making too narrow a cutaneous opening. The combined result of these two surgical faults is that the cutaneous opening closes prematurely to a narrow sinus, the original bubo cavity being left as a pus-pocket, the contents of which finally burrow through the injured abdominal fascia and enter the lymph space.

The cases of immediate death in connection with bubo surgery, wherein the knife of the operator, during the attempt to open the abscess by a single quick incision, penetrates too deeply and lays open a main trunk blood-vessel, are, of course, infrequent. In such cases, as a rule, incision has been attempted without an anæsthetic, and the patient, on the first plunge of the knife point, has sprung upwards from the table, or has struck or seized the surgeon's arm. The fact that many individuals cannot control bodily actions in the event of sudden and severe bodily pain should always be in a surgeon's mind before an attempt is made to open a bubo in this manner; and not only should the arms be held, or so guarded that the operator cannot be seized, but the knife point should be directed superficially or laterally rather than downward, while the end of the little finger of the operating hand should rest, before and during the performance of the incision, on the body of the patient.

Another trouble that has not infrequently arisen has been the deep burrowing of pus, and even secondary hæmorrhage, from erosion of an artery compressed by packing the abscess cavity too drastically with gauze. Professor Fuller lays down the following rules to be observed in opening all buboes:—

In making the primary incision, be careful not to enter the knife-point deeply.

Make the external opening so free that there can be no premature closure of the external wound. In order to be sure of this, a cruciform external incision is often wise, or even necessary.

In the case of a virulent bubo, never curette or disturb the necrotic contents, but allow a natural demarcation to take place between the healthy peripheral tissue and that which is sloughing as the result of the purulent infection.

In the case of suppurative tubercular buboes, after exposing them by free incision, the tumefied glands may be enucleated by means of some blunt instrument, the operator's thumb often being most serviceable for the purpose.

After so removing the glands, do not cut away with curved scissors or with a curette the sloughing, shreddy tissues left at the base, but leave them for a natural detachment.

The cavities of buboes should be but lightly packed with gauze.

